# Forensic odontology in DVI: current practice and recent advances

**DOI:** 10.1080/20961790.2019.1678710

**Published:** 2019-11-06

**Authors:** Alex Forrest

**Affiliations:** a Health Support Queensland Forensic and Scientific Services, Coopers Plains, Queensland, Australia;; b School of Dentistry, The University of Queensland, Brisbane, Australia;; c School of Environment and Science, Griffith University, Nathan, Queensland, Australia

**Keywords:** Forensic sciences, forensic odontology, disaster victim, human identification, mass fatality, radiology, DVI

## Abstract

Forensic odontology frequently plays a significant role in identification of the victims of multi-fatality disasters, but not in all. It depends on adequate dental remains surviving the disaster and on the availability of dental records to be successful. This paper describes current practice in the techniques of identification in forensic odontology and outlines recent advances that are moving into the mainstream.

**Key Points**
- Forensic odontology plays a key role in mass disaster victim identification (DVI) when good-quality antemortem (AM) dental records are available.
- Images including radiographs, computerized tomography (CT) data and three-dimensional (3D) scan data are considered more reliable AM records than written dental charts and odontograms.
- Interpretation, transcription and comparison of dental datasets are complex processes that should be undertaken only by trained dental professionals.
- The future of forensic odontology DVI techniques is likely to include the use of 3D datasets for comparison.

## Introduction

Following a mass casualty incident, identification of the victims may be undertaken in a disaster victim identification (DVI) operation. Visual identification in mass disasters is known to have a high error rate [1] although it is supported by some authors [2]. However, the International Criminal Police Organization (INTERPOL) DVI Guide recommends that all human remains recovered at the scene of a disaster should be stored pending formal identification and release [3]. Early visual identification and cremation or burial of victims such as happened following the South Asian Tsunami in 2004 [4] should be resisted in such circumstances. It can lead to incorrect identifications that cannot be verified and it can subvert the later application of scientifically-based identification techniques. The report of the INTERPOL Tsunami Evaluation Working Group made an explicit recommendation that its DVI Steering Committee reinforced to DVI Standing Committee members that the use of visual identification methods in mass fatality incidents is considered unreliable and almost certainly will lead to the incorrect release of bodies [5]. An incorrect forensic victim identification impacts two parties – those who receive the wrong set of remains, and the family whose loved one will never be identified or returned to them. The DVI process aims to provide a rational, scientific basis for the identification of each victim to ensure that correct identification is assured. It may also seek to link fragments of victims to a previously-identified portion of a body so that the most complete set of remains is returned to surviving family and friends.

The foremost duty of the forensic odontologist in a DVI operation is the identification of unknown deceased individuals. This is achieved by matching postmortem (PM) dental features of a victim with the dental records of a missing person [6] and may require estimation of the age of child victims [7, 8]. It involves the building of a PM dental profile of a victim by dental examination which may include physical and radiographic examination of the teeth and paradental structures, computed tomography (CT) scanning and three-dimensional (3 D) virtual modelling. This PM profile is matched with an antemortem (AM) profile compiled from the dental records of a missing person, which may include written treatment records, images, 3 D datasets, casts of teeth or other dental items that can help individuate a person [9].

Forensic odontological comparison is one of the three principal identifiers designated by INTERPOL for use in identifying the victims of a multi-casualty incident. Its positive outcome is considered sufficient to permit personal identification without further support from other methods [6, 10].

## The role of forensic odontology in DVI

The role played by forensic odontology in mass casualty situations is always the same: comparison of AM and PM dental profiles to determine matches which support identification [6]. The value of the outcome depends on two underlying assumptions: that teeth resist decomposition and relatively extreme environmental conditions (which is established from long observation), and that every person has a set of teeth (a dentition) which is fundamentally and recognisably unique.

The question of whether dentitions are recognisably unique has never been answered [11], just as it has never been answered for fingerprints [12]. The outcome of DNA comparison is also based on the probability of encountering random matches. For this reason, distinctive dental restorations (fillings) increase the opportunities for individuation because they leave artefacts that have unique sizes and shapes that result from the treatment of disease in individual teeth and they can be fabricated from different dental filling materials. Such restorations are radio-opaque when fabricated from metal (dental amalgam or metal crowns for example). Some tooth coloured materials also demonstrate a level of radio-opacity and may fluoresce when exposed to different wavelengths of light [13]. Dental interventions are not limited to fillings alone, but may include dental extractions, implants, prostheses such as full or partial dentures, and a range of surgical treatments. Teeth may be missing because they have failed to develop. The presence of disease or pathology including periodontal (gum) conditions and dental caries (tooth decay), the presence of tooth crowding, or unusual arrangements of teeth in a dental arch and the relationships between teeth in the top and bottom jaws can all add additional features for comparison.

The ability of forensic odontology to add to the forensic identification process in a multi-fatality incident largely depends on the availability and quality of AM dental records [14]. Good-quality dental records are an essential part of patient dental care [15], but not every country has rigorous standards for the documenting of dental treatment and retention of dental records. Particularly in developing countries, the frequent absence of good (or any) dental records may be an impediment to a dental comparison [16]. Disasters in which dental surgeries are destroyed (wildfire, flood, earthquake and tsunami for example) may also impact the availability of dental records [16].

It is almost axiomatic that the greatest numbers of deaths from disasters are likely to occur in countries of low socioeconomic status, possibly due to their inability to invest in preparedness or strategies to mitigate them [17]. In such countries, AM dental records may be of limited availability and debatable quality [16], and so the contribution that forensic odontology can make in DVI operations in those locations or which involve nationals from such regions will be heavily influenced by these factors.

When good-quality AM data are available, forensic odontology classically identifies approximately 60% of victims, and contributes to approximately 30% of further identifications in collaboration with other identifying methods. The usual pattern in a “classical” incident is that the early matches are made by the fingerprints section, followed by a larger contribution from the dental section, with the DNA section providing late results, especially for children without fingerprint or dental records. The DNA section is also able to link fragments to a previously-identified body portion. An excellent systematic review of the role of forensic odontology worldwide in major mass disasters is given by Prajapati et al. [18].

## AM and PM dental profiles

The accuracy and completeness of the AM and PM dental profiles will obviously affect the outcome of any dental comparison. In general, provided sufficient dental remains are present to make construction of a PM profile feasible, its quality is largely a function of the skill, rigour and attention to detail of the examining odontologists. This is under the control of the personnel within the Dental Section and explains why dental data should only ever be subject to acquisition, data entry and data editing by experienced forensic odontologists.

In contrast, members of the Dental Section have comparatively little control over the quality of the AM profile since it is largely reliant on the quality, accuracy and completeness of the AM dental records sourced from hospitals, dental clinics and dental practices. The type and quality of AM records from which the AM profile is compiled will affect the selection of the comparison process(es) to be used and the level of confidence in the overall outcome.

All common aspects of the AM and PM profiles should be considered in determining if the profiles match, so the techniques discussed in this paper are rarely applied in isolation without considering the entire matrix of dental information available for comparison.

## The PM dental examination

It is useful to have access to PM dental radiographs before beginning the physical examination to cross-check observations made in the mouth. INTERPOL recommends taking bitewing radiographs, periapical images of upper and lower molars, premolars and incisors on both sides and separate images of teeth with distinctive features such as root canal fillings or prosthetic dental crowns [3]. When taking periapical images, a parallel technique is recommended rather than a bisecting-angle technique [19, 20]. INTERPOL also recommends PM orthopantomogram (OPG) radiographs [3], but the logistical issue in obtaining these precludes their use in most instances unless special equipment is available. PM OPG images can be simulated by multiplanar reformats of PM CT data if available, and PM CT imaging should be regarded as integral to the PM examination whenever it is available.

The physical dental examination can be very challenging. Rigor mortis may cause difficulty in opening the jaws, and putrefaction can cause leakage of fluids from the soft tissues which can render the teeth difficult to see. Lighting can also be an issue so headset lighting is very useful as are LED-illuminated dental mouth mirrors. Fluids including blood can accumulate in the mouth. Water may be used during tooth brushing to clean the teeth prior to examination and may also build up. When no suction is available, paper towels may be useful to help absorb fluids. Incinerated remains pose particular problems in terms of the friability of remaining teeth and are often best examined by imaging rather than dissection. Tooth-coloured dental restorations may be very difficult to detect in PM circumstances, but may fluoresce when illuminated by alternate light sources [21]. Trauma can result in lost teeth lying in a body bag which may not be noted by the dental team. INTERPOL recommends that jaws should not be removed unless absolutely necessary and then only with the approval of the controlling authority with the provision that they are kept with the body at all times [3]. Photographs, including images of the occlusal surfaces, should be taken to document the teeth thoroughly. INTERPOL provides excellent guidance on PM photographic documentation [3].

When performed according to INTERPOL guidelines, it is normal for odontologists in the PM team to work in pairs so that one can record the observations while the other performs the examination to reduce contamination. They should subsequently rotate positions and repeat the exam, cross-checking the results of their examinations and then transcribing them onto the appropriate forms for quality assurance [3]. The transcription (or computer record in case of direct data entry) should also be cross-checked. The outcome should then be subject to at least one further quality assurance process before being entered into the pool of PM profiles available for comparison.

The final PM dental profile should contain complete written documentation for every tooth whether it is present in the mouth or not, together with a corresponding completed odontogram as well as complete radiographic and photographic documentation.

If paper forms are used, they will now need to be decontaminated. If a computer is used, the process of subsequent manual data entry should also be undertaken by teams of two forensic odontologists for quality assurance because this extra stage provides an opportunity for further errors to be introduced.

## AM dental records

### Written dental records

Written dental records are among the least useful of all dental records and may be described as a surrogate record [22]. They are considered a subjective record because they do not derive directly from a patient and much of what they record is interpreted by a dentist during examination, treatment, or by a scribe during transcription.

**Figure 1.**
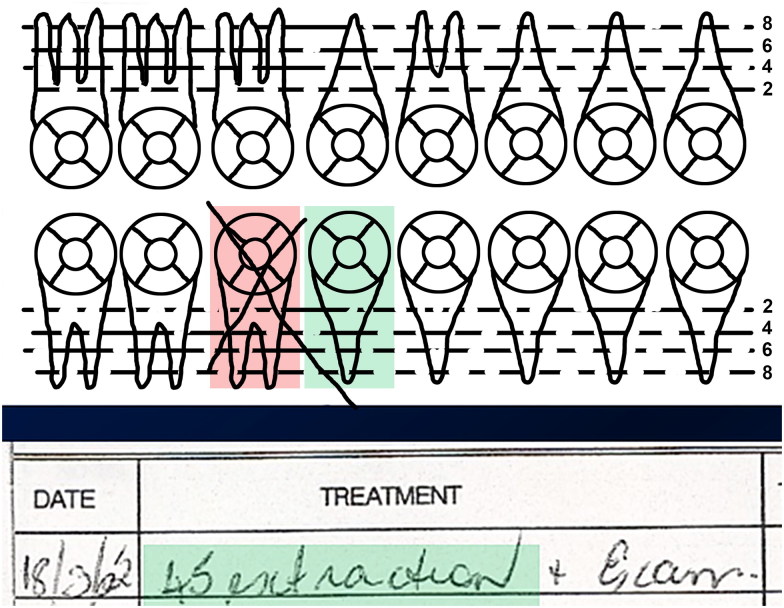
In the dental record, tooth 45 (lower right second premolar, shaded green in the odontogram and treatment notes) is recorded as having been extracted, but it is tooth 46 (shaded in pink) that has been crossed out. We cannot resolve this problem without further information or images such as X-rays. (Image courtesy Health Support Queensland Forensic and Scientific Services (HSQ FSS) and the Queensland State Coroner, with permission).

Forensic odontologists often receive dental records that are poorly documented [23], incomplete, or difficult to understand [24]. They are written accounts of observations made on a patient or of treatment carried out on a patient. They may fail to record items required by professional record-keeping guidelines that are of significance to the forensic odontologist [14]. The portions recording items of dental treatment and the dates on which they were performed are known as Treatment Notes and may or may not be accompanied by numerical codes which may differ from one jurisdiction to another indicating specific treatments. Observations made during a dental examination or following treatment may have been recorded by a third party. A dental surgery is a noisy environment, so in this circumstance the recorder will write down what they thought they heard the dentist say, and what they thought they saw or did [22]. Errors may therefore occur during the transcription and they may also occur through carelessness or inattention [24]. As both a failsafe and allowing for rapid assessment of a patient’s status, many written dental records also feature an odontogram. An odontogram is a stylized diagram of the teeth on which planned and executed dental interventions can be recorded in a visual format. Obviously, this ought to accord with the information in the treatment notes. Figure 1 shows a portion of a genuine dental record in which the lower right first molar tooth is shown as extracted in the odontogram (crossed out), but the lower right second premolar tooth is recorded as extracted in the treatment notes. This is an example of an error that, without further information, renders the written record useless. However, the danger is even more subtle; it is also possible that both the treatment notes and the odontogram will concur, but both will record that the wrong tooth has been treated. This type of error cannot be detected when using written records alone and may result in an inadvertent exclusion of identity. Experienced forensic odontologists exclude a potential match very sparingly and carefully if only written dental records are available.

The protocols for specifying teeth also vary. The most commonly used system is known as World Dental Federation (FDI) notation, and this is recommended by INTERPOL for use in national and international DVI operations that follow INTERPOL DVI Guidelines [3]. In this system, the mouth is divided into four quadrants; the upper right quadrant is indicated as quadrant 1, the upper left as quadrant 2, and so on clockwise around the mouth. Within each quadrant there are eight teeth in a fully dentate adult mouth, and they are numbered 1 to 8 from the midline backwards. Thus, the upper left first molar tooth (tooth 6 in the upper left quadrant – quadrant 2 – is numbered 26 (pronounced two-six)). This contrasts with the Universal (or National) System in which the adult teeth are numbered clockwise from the backmost tooth on the upper right clockwise around the mouth. In this system the upper left first molar tooth (14th tooth along from the back on the right) would be tooth 14, pronounced “fourteen”. A further system in common use is the Palmer System in which the adult teeth in each quadrant are numbered from 1 to 8 working from the midline backwards and this system uses a grid symbol to indicate the relevant quadrant [25]. Confusion could arise in a poorly-handwritten record. Could what appears to be tooth “47” carelessly written in Palmer notation mean the lower right first premolar?

None of these systems has gained full international acceptance and none of them copes with supernumerary teeth [24]. A forensic odontologist must know which system has been used when interpreting AM dental records received from different jurisdictions or countries.

There is currently no standardised way of expressing dental terminology unambiguously from one country to another. At the time of writing, the International Standards Organisation (ISO) Technical Committee 106, the committee which deals with dental terminology, has created a working group known as “ISO/TC 106/SC 3/WG 5 – Terminology for Forensic Oro-Dental Data”. This committee comprises members from 24 countries and includes official liaison personnel from INTERPOL and the North Atlantic Treaty Organization (NATO). Its mission is to codify recommended terminology across different countries so that forensic dental data can be communicated in a consistent fashion and it expects to report shortly (Dr. Kenneth Asccheim, personal communication, unreferenced).

### Dental radiographs (X-rays)

Radiographs (X-rays) are a more reliable form of dental record. They record the shadows of objects which are cast against a special sensor when they are illuminated with X-radiation and are usually shown as a negative (radiopaque objects shown white). They are an objective record, which is to say they record exactly what is present in a visual way [22, 26]. This means they are immune from the subjective recording and interpretation issues that plague written dental records and so they can be used to confirm the information that written records contain. They may also show tooth-coloured fillings that might not be apparent in the mouth [19]. They are therefore a valuable resource, but they are two-dimensional (2 D) and should be used *in conjunction* with written records which may contain additional information about treatment performed after the X-ray was taken. The written record can be used to determine whether a filling that appears on the side of a tooth in an X-ray is on the side closest to the lips and cheeks or on the reverse side. They may reveal information that is not visible from a dental examination in the mouth, for instance the presence of root canal fillings or the presence of tooth-coloured restorations [19]. There may be some difficulty in confidently determining the correct orientation (left or right) with some AM digital intraoral images [27].

### CT data

AM CT scan data are a very valuable resource. They may originate from a medical CT scanner or from a Cone Beam CT (CBCT) machine which may be installed in a dental office or a radiology practice. CBCT scans usually feature fewer beam hardening artefacts and less soft tissue information than medical CT scans [28, 29]. Beam hardening is caused by high atomic number materials such as metal amalgam and implants. These materials filter out lower energy photons in the X-ray beam to “harden” it and Compton scattering causes streaking which obscures the surface detail of the material [30]. However, regardless of the machine used, the data capture 3 D information about the scanned portion of a patient and can be viewed in different ways. It can be used to create a virtual 3 D model of the teeth and bones (which can be actualised using a 3 D printer if necessary [31]), viewed as a simulation of a 3 D X-ray, or sliced in such a way as to simulate various dental X-rays including the OPG – a view that records the entire dentition on a single image [19].

The CT is one of the most versatile of all imaging modalities used in forensic odontology. Because of its 3 D nature, it can resolve questions about which side of a tooth contains a filling and it can show distinctive anatomy from many different angles. The 3 D reformatted view also respects perspective when zoomed in and out in some systems, making it a valuable tool when attempting to superimpose an image of the teeth of the deceased person over a photograph of a missing person.

### 3D Surface scan data

3D scanners are replacing conventional dental impression techniques [32]. During the latter, a suitably-sized tray containing a thick paste is inserted into a patient’s mouth and both the dentist and the patient must wait for the material to set before it can be removed to show a space representing the teeth and gums. This can be a traumatic process for both the dentist and the patient, particularly in patients with a trigger gag reflex. Recent 3 D intraoral scanners comprise a wand connected to a computer passed over the teeth (or other item) and it records data which are processed in real time to produce an accurate 3 D virtual model. This can also be actualised with a 3 D printer. These scans are clinically accurate [32, 33], and the process is very comfortable for the patient, especially if a powder-free technique is used. As they become increasingly widespread, 3 D surface scans comprise a new set of AM data. They do not rely on ionising radiation (unlike X-rays and CT scans) and are not affected by the presence of prior dental treatments. Unlike CT data, they are equally useful regardless of whether the teeth contain fillings (of any material) or not.

### Dental study models

Study models are classically poured from dental impressions in hard gypsum-based materials. They are 3 D casts representing a dentition, and they are extremely valuable because they function as a proxy for the patient. Their surfaces can be recorded using a 3 D scanner and compared with a similar scan of the teeth of a deceased person.

### Dental appliances

Dental appliances may include such items as full or partial dentures, orthodontic appliances, occlusal splints, bleaching trays and mouthguards. All of these may be useful for comparison with the dentition or mouth of a deceased person. Not all such appliances need to be made in a dental surgery. For example, mouthguards may be fabricated in schools for students who play contact sports and may also be specially made for sports teams, and home mouthguard construction options are available. Partial dentures are especially valuable as they are made to fit a single mouth. Sometimes, dentures may be marked with a unique patient mark or number, particularly in hospices and nursing homes [34].

### Clinical photographs

Clinical photographs may record teeth of a patient in some detail. They can be useful for comparison with similar photographs of the teeth of a deceased person. It should be borne in mind that duplicating AM photographs in a PM situation can present difficulties, however. Cadavers are not generally cooperative, and decomposition, incineration or rigor mortis can present serious problems.

### Photographs showing smiling faces

Recently, it has become common for individuals to post smiling photographs of themselves on social media, which may therefore represent a source of AM dental information. It may be possible to superimpose a PM photograph of a victim’s teeth over the teeth shown on a photograph of a missing person. However, images on social media are commonly of low resolution. It takes a great deal of skill to accurately replicate the camera distance and position when securing a PM image for comparison although protocols are available [35], but cadavers are not cooperative. In the case of an AM professional portrait image, the camera may need to be placed as much as 2 m away from the teeth which can be difficult in a mortuary situation. It may be simpler to use data from a 3 D reconstruction from CT or from a 3 D scanner [36] to achieve a result.

Absent from this list at the time of writing is the comparison of serial numbers etched into dental implants. They are absent for a good reason; regrettably dental implants mostly do not yet feature serial numbers. Identifying the type of implant(s) present may help to reduce the number of potential matches, and an excellent website (https://whatimplantisthat.com/) provides a comprehensive catalogue of radiographic and graphic implant profiles against which PM implants can be compared. However, implants are subject to counterfeit, so the comparison process is still subject to caveats on that basis.

## Computers as an aid to forensic comparison

When substantial numbers of victims are involved, the use of a computer programme to help in the comparison process is a very useful (if not vital) requirement. Many such computer programmes exist, some of which are dedicated solely to comparison of dental data [37]. They may operate on the basis of simple AM and PM profiles that record only a subset of possible dental data (such as whether a tooth is decayed, filled, sound or absent) or may utilise comprehensive dental information. One of the most widely used of these is DVISys (dvi@plass.dk), developed by Plass Data, a company based in Denmark. At the time of writing, 194 countries are INTERPOL members and therefore nominally subscribe to INTERPOL protocols in DVI. DVISys reflects the INTERPOL yellow (missing person) and pink (unidentified human remains) forms and accords with INTERPOL protocols. For this reason, it is the most widely used system worldwide and also one of the most comprehensive, with dental comparison comprising only one of several components. It has been proved effective in several major DVI operations at the time of writing [38], as well as being used in missing persons comparisons and routine forensic comparison casework.

Like all computer programmes that manipulate data, it depends on the quality of information entered. Erroneous or poor-quality records, whether AM or PM, will inevitably result in poor outcomes. For this reason, it ranks possible matches for subsequent human scrutiny. Interpreting the ranked matches is a role for an experienced forensic odontologist. Use of a computer programme such as this can lead to successful outcomes very rapidly when good quality data are available.

There comes a point in any large DVI incident when any programme reaches the limit of its data and can no longer be useful. It is important to recognise this point, which indicates either the need for more (or better) data, or a change of strategy, or both. In such a case, it has been recognised that a process involving progressive filtration of cases by successive expert groups may be useful. For example, during the DVI operation in Thailand following the 2004 South Asian Tsunami, the DNA group would provide a series of partial PM matches for a given missing person and pass them on to the Dental group, whose members would attempt to eliminate as many as possible on dental grounds. If identification could not be confirmed at that stage, cases remaining were then passed to the Fingerprints team for examination. If insufficient data were present for any of these teams to make a case, the files were passed to an Investigative team with targeted requests for additional information which tightly focused the new investigative efforts [39]. In this sense, DVISys was useful as a filter for cases that could not be advanced by the computer matching strategy and therefore required a different approach.

## The comparison process

The type of comparison performed depends on the nature of the DVI incident (closed or open), the type and quality of the AM dental records available, and the facilities available for PM dental examination and imaging. It may also involve the use of a computer programme to provide a series of most likely matches for the Forensic Odontologist to consider.

### Written dental records

If dental records are sourced from another location or country, particularly if the language there is different from the one in the country in which the DVI operation is happening, it can be very advantageous to have forensic odontologists from that other country perform transcription of the dental records onto the INTERPOL missing persons form. Local odontologists are more likely to be familiar with local charting conventions, and this can resolve confusion during the transcription process. The transcription should be in the language currently being used in the DVI operation and it should be cross-checked by at least one additional local odontologist for quality assurance purposes.

AM dental records are not always recent or complete (the patient may have visited multiple dentists but records from only some of them may be available) and therefore forensic odontologists do not always seek a perfect match between the AM and PM dental profiles. Instead, they ask if the PM profile could have evolved from the AM profile because further dental treatment may have been provided by different dentists since the last available treatment record. Generally, there should be no unexplainable discrepancies between the AM and PM dental profiles before a case is made for identification on dental grounds [9].

### 2D Images

**Figure 2.**
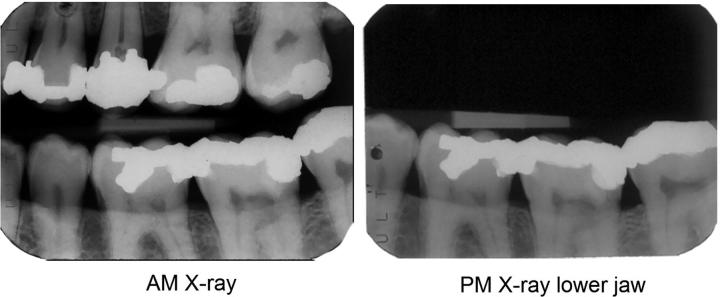
X-ray image comparison. The bright white areas in the tooth crowns represent the unique shapes of metal amalgam fillings. The chances of getting such a level of similarity between the teeth, filling shapes and bone levels in two individuals chosen at random would be exceedingly small, so it is reasonable to state that these images originate from the same person. (Image courtesy Health Support Queensland Forensic and Scientific Services (HSQ FSS) and the Queensland State Coroner, with permission).

Comparison of AM and PM 2 D images such as X-rays (Figure 2) provides a level of confidence that cannot usually be achieved by using written dental records alone unless there are multiple highly-probative concordant features in them with no unexplainable contradictions [19]. Because the images are objective records of the dentition at the time they were made, their comparison is rarely ambiguous if dental restorations are present but may present difficulties if no radio-opaque restorations or individuating features are visible [26]. Several studies have attempted to validate the process, but the absence of a consistent methodology between them makes it difficult to draw meaningful overall conclusions [40]. Experience in DVI incidents where identification is established by multiple methods including comparison of AM and PM dental X-rays demonstrates that the technique is reliable.

**Figure 3.**
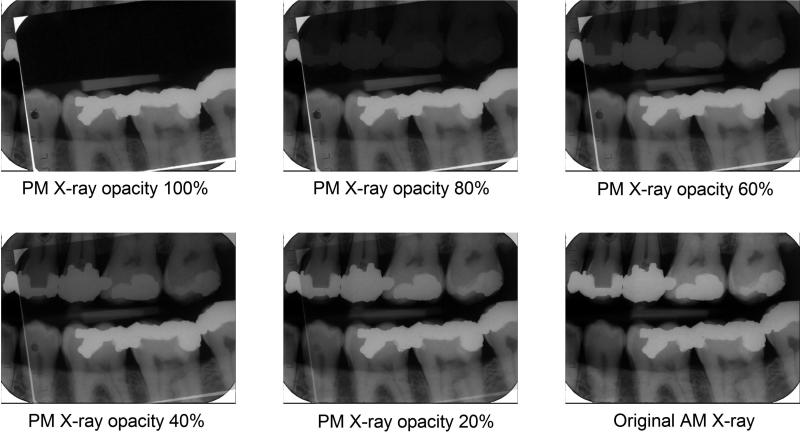
X-ray superimposition. Superimposing the images demonstrates the similarities between the dental features in an intuitive way. (Image courtesy Health Support Queensland Forensic and Scientific Services (HSQ FSS) and the Queensland State Coroner, with permission).

This comparison process can be taken to another level by superimposing the two images [26] as in Figure 3.

**Figure 4.**
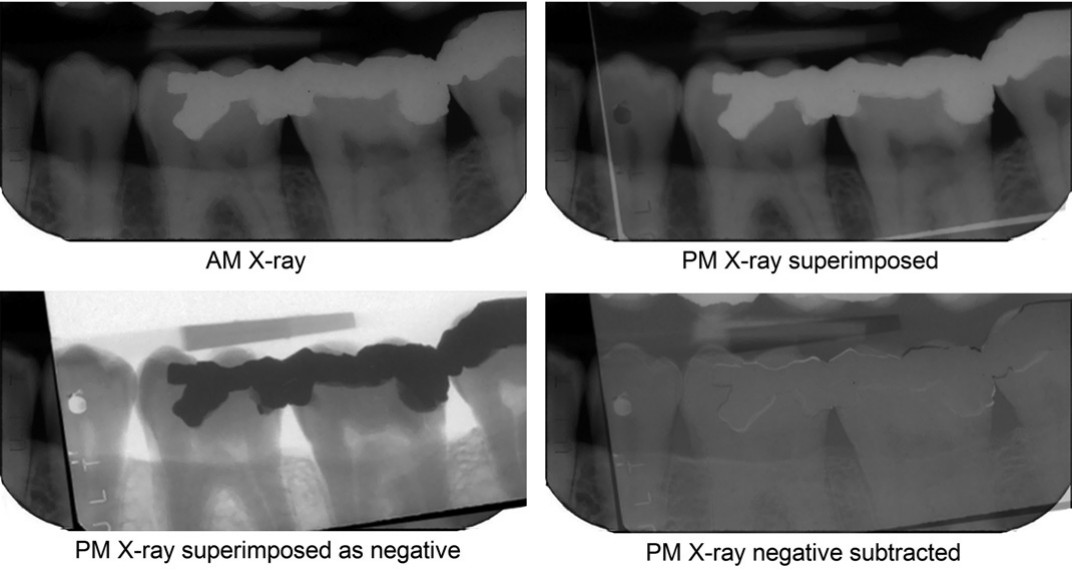
Subtraction imaging. The postmortem (PM) image is superimposed on the antemortem (AM) image and turned into a negative. When its opacity is reduced, the AM image becomes progressively more visible until all the overlying negative and underlying positive colours cancel to resolve to grey wherever the images are similar. Changed features now become clear. The suggestion of a white margin around the amalgam fillings indicates a slight mismatch in the sensor or beam alignment or both between the two images, demonstrating that they are the same object. (Image courtesy Health Support Queensland Forensic and Scientific Services (HSQ FSS) and the Queensland State Coroner, with permission).

Finally, subtraction imaging can be used to prove that the two images originated from the same source [22] as shown in Figure 4.

The level of skill required in taking PM X-rays that are sufficiently similar to their AM counterparts to allow for the possibility of superimposition and subtraction images is considerable. It will usually require the taking of subsequent PM X-rays after the AM images have been received.

These techniques are useful to apply in small closed disasters but may be logistically complex in larger incidents.

### CT and CBCT images

CT images are extremely useful. They can be acquired using a mobile scanner, and without opening a body bag or disturbing the remains [41] which may be critical following incineration [29, 42]. They can reveal the state and morphology of the dental remains prior to the actual viewing of the deceased and help determine if and where loose teeth may be scattered inside a body bag. Critically, they can reduce the incidence of removal of lower jaws as part of the PM dental examination process since details of the teeth from both jaws can be viewed without the remains being disturbed [43]. The data can be segmented and reformatted in different ways to produce different views of the scanned dentition including 3 D visualizations of hard tissues, simulations of 3 D X-ray images, or various dental 2 D images [20, 28].

Unfortunately, high attenuation objects such as amalgam restorations produce beam-hardening and photon starvation which result in images that show characteristic streaking artefacts [44]. Numerous methods and algorithms have been implemented to reduce these [45]. The detailed morphology of such metal items is generally not as well demonstrated in CT images as it is in plain film X-rays, so when comparison or superimposition of AM and PM images is to be performed, it is often better to secure plain film images of a deceased person for the purpose. The CT data can be used to inform the forensic odontologist about the state of the teeth of the deceased so as to secure the most useful images. The irony is that the scans which demonstrate the best morphology are usually those without metallic fillings, but the most useful are those with metallic fillings which are most affected by artefacts. There are many other morphological details that can be compared with OPG X-rays including tooth root and pulp shape, sinus morphology, patterns of tooth crowding, missing teeth and periodontal bone levels as well as bony pathology. This is fortunate since decreasing numbers of young people feature metal restoration. Victims with large numbers of metallic restorations can be also subjected to 3 D surface scanning which operates equally well regardless of the presence or absence of metal.

CT images may be used in several ways by forensic odontologists. These include direct comparison of CT slices, which is especially useful in comparing the morphology of pneumatic sinuses including the maxillary sinuses, ethmoid air cells and frontal sinuses. It is important to ensure that the PM and AM slices are taken with precisely the same plane orientation, and this requires experience, but may be aided by using multiplanar reconstructions (MPR) to determine slice orientation [46]. The aid of a radiographer or radiologist is very helpful when using this technique. CT images have also been used to differentiate between dental materials used in tooth-coloured fillings [47] although this is only useful if the material has been recorded in the AM dental record.

Another technique is to perform MPR of the CT or CBCT data to simulate common dental X-ray images [28]. They can be used to simulate dental periapical images and OPG X-rays. The thickness of the plane to be reconstructed is an important consideration. Once the path of the reconstruction has been determined, it is useful to increase the thickness of the slice on either side of the central plane or path. While thicker slices allow the capture of more information, they are also affected by poorer image sharpness. The optimum slice thickness is case-dependent and relates to the data that need to be visualised. A slice thickness of 10 mm – 15 mm seems to return the optimum balance between information and image sharpness in most cases. Because the orientation of the jaws may be different following death due to trauma or displacement, it is often necessary to perform separate MPR for upper and lower jaws as they may not remain in the same relative positions as they occupied during life. Fragmentation may also require different fragments to be reconstructed separately.

**Figure 5.**
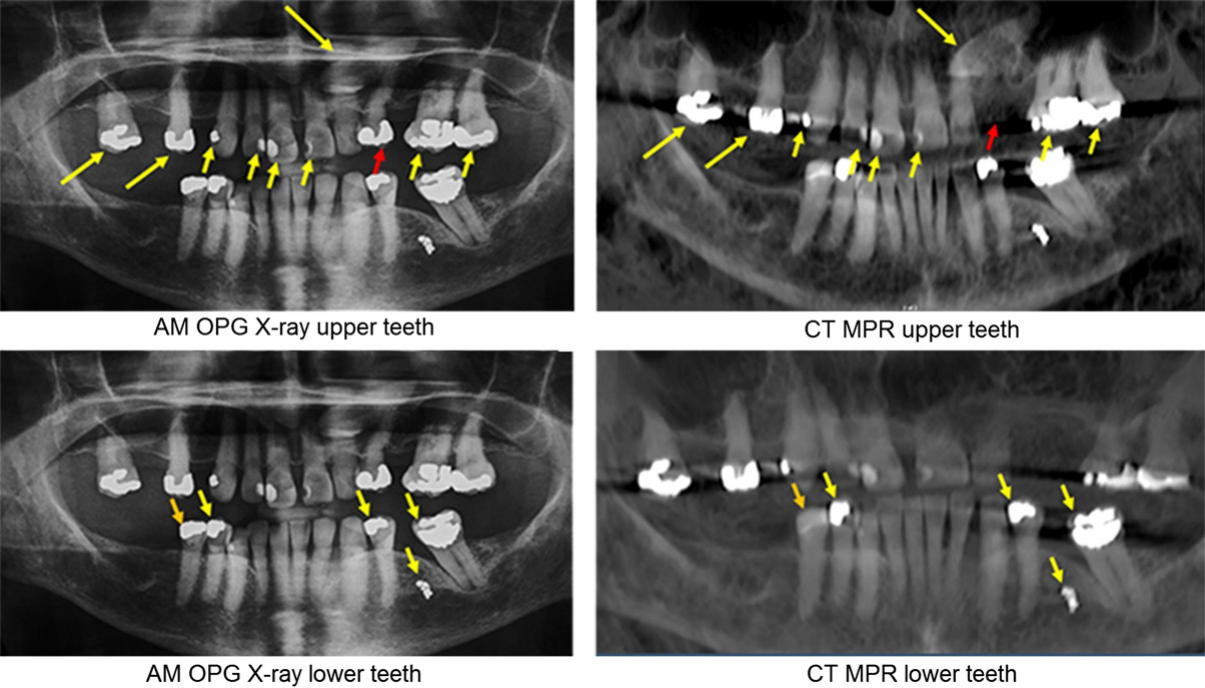
Comparison between antemortem (AM) orthopantomogram (OPG) X-ray of a missing person and multiplanar reconstructions (MPR) of CT data of the upper and lower jaws of a victim. Corresponding features are indicated by yellow arrows. The red arrow in the upper jaw indicates a tooth that was extracted before death and the orange arrow in the lower jaw indicates a filling that was replaced. Both are accounted for in the written dental record. (Image courtesy Health Support Queensland Forensic and Scientific Services (HSQ FSS) and the Queensland State Coroner, with permission).

The images resulting from reconstruction can be directly compared with AM intraoral or OPG images (a technique that has been previously validated [48]), or they may be used to guide the forensic odontologist as to which PM plain film images should be captured for later definitive comparison with AM X-rays. Direct comparison between AM OPG and PM reconstructed images is most useful when many distinctive dental interventions are present as shown in Figure 5.

Where children are among the victims of a small DVI operation, it may be useful to use the CT images to rank them according to increasing age on receipt of the CT data rather than performing formal age estimation which can be undertaken later. This may help in targeting the identification effort and providing information about whether useful AM dental records might be available. While CT and MRI images may be useful for age estimation, more research needs to be devoted to this topic [49].

### 3D Surface scan data

3D dental impression techniques are beginning to make inroads into dental practice. While they are appearing in the general dental practices in developed countries, orthodontic and prosthodontic specialist practices have been among the early adopters, so it is important to consider such specialist practices when seeking AM dental records. Information about specialist referrals should always be sought as part of the AM record investigation. Dental laboratories are increasingly becoming equipped to handle digital workflows in developed countries, which in turn encourages general dental practitioners to adopt digital impression techniques. The equipment required is improving rapidly and it is becoming more affordable and better supported. Practices are also finding that they no longer need storage space for large numbers of physical dental models when virtual models can be stored digitally.

**Figure 6.**
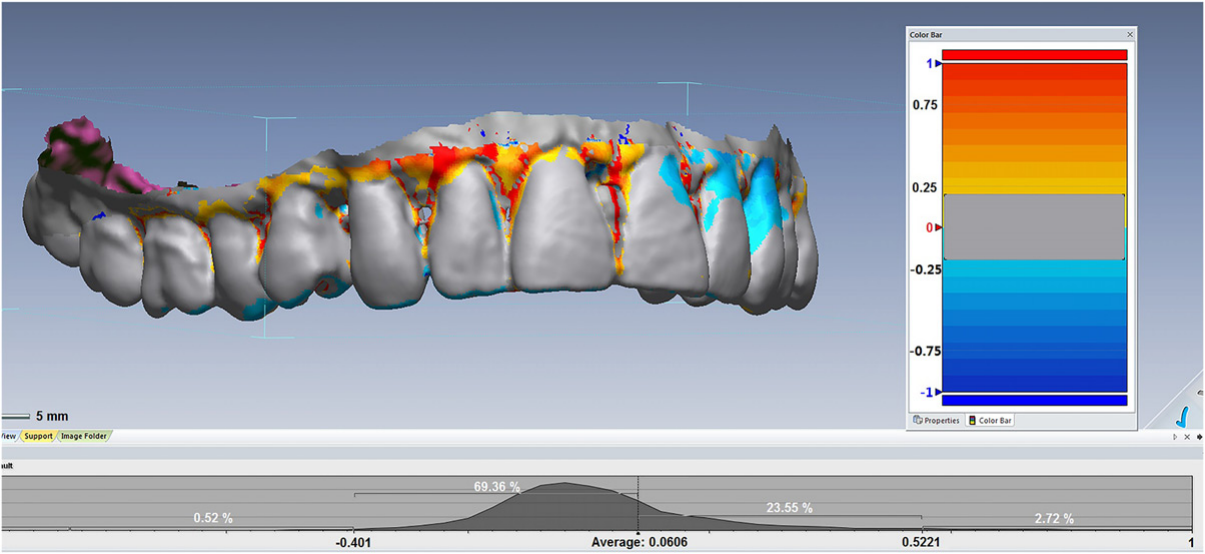
Three-dimensional (3 D) superimposition. The postmortem (PM) surface is superimposed on the antemortem (AM) surface. In this case, scans were secured by a low-resolution medical CT scanner with an approximately 0.3 slice thickness. Surfaces matching within a region of 0.5 mm are coloured grey, while those that fall outside this range (mainly soft tissues) are coloured according to the degree of mismatch. With modern intraoral scanners, the resolution and scan precision are much higher, so the width of the grey zone can be reduced to a level measured in tens of microns, providing excellent individuation. (Image courtesy Ms Donna MacGregor and Mr Mark Barry, with permission).

When AM 3 D surface scan data are available, they can be compared roughly with reformatted surface data from CT or CBCT PM scans (Figure 6), or more accurately with PM data from a comparable 3 D scanner. It is likely that the technique of 3 D comparison will become increasingly important for use in single-case identifications and this process potentially can be automated for use in DVI operations. In the future, 3 D intraoral scanners will become a routine tool in the forensic odontologist’s armamentarium as high-resolution 3 D AM data become increasingly available.

Comparison of 3 D scans of palatal rugae for identification has been suggested [50, 51], including for differentiating between monozygotic twins [52]. There are conflicting views as to whether orthodontic treatment changes the morphology of palatal rugae sufficiently to compromise their use for identification [53, 54].

Questions still remain to be answered. The utility of full-arch 3 D scans is obvious, especially if both upper and lower AM arch scans are available. Scans of smaller sections of a dental arch are likely to be performed by dentists using the data for the construction of dental restorations such as crowns or implants, both of which involve changing the shapes of teeth or replacing missing teeth during treatment. The feasibility of using these scans of smaller segments to compare with PM data is still not known, particularly after the dental intervention is completed. To what extent does major dental restorative work affect the outcome? Data are not yet available on the results of comparing orthodontic scans (a specialty that routinely scans entire dental arches) when the teeth are moved during treatment. The smallest fragment size that can be used to confidently identify an individual using this technique is not known, and since some disasters involve fragmentation of the jaws, this is an important question to answer. A great deal of research needs to be undertaken to answer these questions, but it seems clear that this third dimension will be a major focus of forensic odontology in the future, especially if the comparison process can be automated and incorporated into a programme such as DVISys.

### Removable dental appliances

Of all the removable dental appliances, metal-framed partial dentures provide the most distinctive information. They are precision-made to fit a single mouth and may provide a compelling method of identification if their AM provenance can be demonstrated. If there is no information about the identity of the person to whom they belonged in life, their presence is not helpful. Mercifully, they are occasionally found in the home of a missing person in which case the identity of the owner may be inferred. Acrylic partial dentures are also very useful if they can be fitted to a victim’s teeth, but do not generally fit with the same precision as those made with metal frames.

Full dentures are less useful. In the event of decomposition or fragmentation, an opinion as to their fit is more likely to be exclusionary than inclusionary.

Dentures may be marked with identifying information, and this is most often the case if victims have been living in a nursing home or similar establishment. Numerous methods have been proposed for marking dentures over the years, but it is still far less common than forensic odontologists would wish [55].

Mouthguards may be available for younger victims. While these are made of pliable material and are not precision appliances in the sense that metal-based partial dentures commonly are, they may be useful because they represent a reverse model of the teeth they are designed to fit. While they can be fitted directly to the teeth of a victim, a better demonstration is to use them as moulds to create a low-resolution physical model of the teeth they were made from, and such a model can in turn be compared with the dentition of a deceased person by making a further mould from it, observing its fit on the teeth of the victim, and slicing it with a scalpel if necessary to demonstrate its adaptation to the tooth surfaces. This method was used at least once in the identification of a victim of the 1st Bali Terrorist Bombing in 2002.

More recently, mandibular advancement splints have become increasingly common in the treatment of sleep apnoea, and may serve a similar purpose to mouthguards, usually with slightly better precision since they are rigid structures. They are fabricated to cover teeth of both jaws and may therefore provide more individuating information than mouthguards unless they, too are designed to stabilize both jaws (bi-maxillary mouthguards).

Orthodontic appliances can also be compared to the teeth of a victim to determine their fit.

### Radio-frequency identification (RFID) tags

RFID tags have been proposed as aids to personal identification and were trialled in some victims in the South Asian Tsunami DVI operation in 2005 to supplement printed identification labels [56] and following Hurricane Katrina in the US [57]. They may be implanted into teeth [58] or into removable appliances such as dentures [59]. Because their implantation into teeth is an invasive procedure and requires the preparation of a cavity in healthy tooth structure with a subsequent dental restoration, it has not gained widespread acceptance. Using filling materials of a different colour to signal their presence has also been proposed. RFID technology was also used for body tracking in the Hurricane Katrina DVI operation [15].

## Advantages of digital technologies in forensic odontology

AM digital dental records may include computerised dental record systems which record both dental treatment and an odontogram for a patient, replacing the physical record. Plain film X-rays including OPG images are routinely captured as digital images in developed countries, and CT, CBCT and 3 D surface data are digital by their nature. Corresponding PM data can and should be digital in nature whenever possible, and chemical X-ray film processing should be avoided as it must subsequently be digitised for storage in a computer which introduces possible quality issues. The requirement for a dentist to label physical X-ray film with the identity of a patient or a forensic odontologist to label a film with the identification number of a victim after processing are two further potential sources of error.

The major advantages of digital data are that they can be stored for very long periods of time on increasingly capacious storage technologies. They can (and should) be routinely backed up, including to secure cloud-based facilities, to ensure their continued integrity and permanence. Off-site backup also ensures that they are less likely to be lost if a dental practice or facility (or mortuary) is destroyed.

Digital data can be transmitted very rapidly and are received with identical characteristics as when they were transmitted; there is no loss of resolution or quality. They are immediately useful upon receipt; images do not require further scanning or imaging as do physical X-ray films, and because original films or dental models are not being sent, identical copies remain at source so that records are less likely to be lost during the DVI procedure (which should also include a data backup strategy). Image data can be directly imported into a computer programme such as DVISys, reducing one possible source of error. The use of digital dental PM data recording is a further opportunity to ensure data are entered directly into a computer programme such as DVISys, eliminating a source of transcription error.

Extensive use of digital data and data transmission requires robust access to the Internet at the DVI Victim Identification Centre, and this may not be easily available in developing countries or in remote locations. DVI planning needs to take this into account when formulating their strategy for handling AM data.

## When is forensic odontology useful in DVI incidents?

Dental identification techniques are dependent on the presence of adequate dental remains, and on the availability and quality of dental records.

It is notable that in some developing countries, dental records may not be routinely completed for dental treatment [16]. As dentists move into the forensic arena in those countries (a process which is now becoming widespread and accelerating thanks to conferences, meetings and ease of consultation with fellow specialists through restricted social media threads), recognition among dental practitioners, local dental associations and government bodies to ensure that dental records are recorded, maintained and kept is being promoted in the literature [18].

Unfortunately, even in some major developed countries, legislation or by-laws only mandate the retention of dental records for a limited number of years [60, 61]. Since dental records (including images) are increasingly digital in nature, and since storage capacities are increasing at a very rapid rate, it is not clear why this cannot be addressed to increase retention times indefinitely. The issue of storing physical records and dental study models is a vanishing problem for those who have migrated to digital technologies, and this is a trend that can only continue. Further, digital records can easily be backed-up offsite, rendering them less susceptible to destruction if a dental practice is physically destroyed during a disaster.

## Future directions

It seems that the future of comparison technologies in forensic odontology is firmly based in the third dimension. As CT and possibly CBCT become more tightly integrated into PM examination, 3 D datasets of teeth, tooth-bearing fragments and dental arches will become available even before a body bag is opened. As digital impressions begin to move into the mainstream of modern dental practice, AM 3 D datasets will be increasingly part of the AM dental profile, and 3 D intraoral scanners will become indispensable parts of the forensic odontologists toolset. All of these converge at a point where AM and PM 3 D virtual models of a dentition will become available.

At present, there is a dearth of affordable easy-to-use public-domain software running on multiple platforms to permit easy and quick 3 D object comparisons from multiple different imaging modalities, although programmes like MeshLab [62] offer opportunities for comparison of point clouds as well as meshes. It is to be hoped that such simple software emerges.

It is possible that in the future, the process of 3 D comparison could be automated, providing opportunities for very rapid and reliable AM and PM model matching.

## Conclusion

The purpose of the DVI process is to provide a rational, scientific basis for the determination of the identity of victims in a mass-casualty incident.

Each disaster is unique, so no universal statements can be made about the routine usefulness of any of the principal identifiers. Fires, trauma or environmental factors may destroy limbs and digits, compromising the contribution of ridgeology. DNA profiling may be affected by incineration or environmental factors and is time-consuming and expensive. It depends on the availability of AM profiles with which to compare that of a deceased person but may also be able to link fragments to a previously-identified body portion. Forensic odontology may be affected if dental remains are destroyed or not found, or when dental records are inadequate or not available.

Where dental remains and adequate AM dental records are available, forensic odontology can be expected to make a large contribution to the identification effort. Excellent techniques and technologies are currently available to the forensic odontologist to ensure accurate outcomes and to demonstrate the basis of their conclusions to an Identification Board.

New technologies and tools are rapidly developing. A decade ago, CT was a new technology being applied in this field, and 3D surface comparison was an emerging method not yet used routinely. Both technologies depend on the increasing adoption of 3 D imaging in routine dental and medical practice to ensure that objective AM data are available for comparison with the dental features of victims. CT scanning is now a routine medical procedure, and 3 D surface scanning of teeth to create digital dental impressions is becoming more widely adopted, particularly in orthodontic and prosthodontic practices and in maxillofacial surgery. This emphasizes the importance of seeking dental records from specialists as well as from general dental practitioners.

Digital data can be easily and rapidly transmitted with no loss of accuracy or detail, and this can greatly speed a response in a DVI operation.

The future of forensic odontology in DVI will increasingly depend on 3 D datasets including CT and 3 D surface scan data, and current forensic odontology practitioners will become progressively more experienced in the utilisation of these technologies as they become more tightly integrated into daily practice.

Developing countries are increasingly recognising the importance of forensic odontology in routine casework as well as in DVI, and practitioners in many of those countries are attending forensic odontology conferences and meetings and acquiring the skills and equipment needed to provide a forensic odontology service. Their activities should be promoted and supported by the forensic odontology and general forensic communities at large to ensure that capacity for a scientific response is built across the globe.

## Author’s contribution

The author was the sole contributor to this paper.

## Compliance with ethical standard

This article does not contain any studies with human participants or animals performed by the author.
